# Enteral glutamine supplementation in critically ill patients

**DOI:** 10.1186/s13054-015-1111-6

**Published:** 2015-11-24

**Authors:** Xu Tian, Guo-Min Song

**Affiliations:** Graduate College, Tianjin University of Traditional Chinese Medicine, Anshan West Road, Nankai District, Tianjin 300193 China; Department of Nursing, Tianjin Hospital, 406 Jiefang South Road, Hexi District, Tianjin 300211 China

In *Critical Care* recently, we read with great interest the systematic review and meta-analysis by van Zanten and colleagues, who compared enteral glutamine with control in critically ill patients in terms of clinical outcomes [1]. We enthusiastically congratulate and applaud them for their groundbreaking work on this topic. However, some issues in their study should be noted and further discussed. Firstly, a predefined subgroup analysis was adopted through excluding the randomized controlled trials (RCTs) done by van Zanten and colleagues, in which other supplemental nutrients were used [2]. To test the robustness of overall effects, these authors performed this sensitivity analysis appropriately. However, a sensitivity analysis was not performed in all subgroup analyses. Although subgroup analysis is one of the methods of sensitivity analysis, it should be carried out in order to further test the influence of an individual study on the overall results [3]. Secondly, the variations of each mean difference reported in all eligible RCTs are so obvious in terms of intensive care unit length of stay and thus a standardized mean difference (SMD) effect size rather than weighted mean difference should be selected to measure the pooled result, even though SMD does not resonate well with most clinicians [3]. Thirdly, these authors stated that “a unique feature of this meta-analysis is that no language restrictions are placed on the searches” [1]. However, it should be noted that only EMBASE, MEDLINE, CINAHL (Cumulative Index to Nursing and Allied Health Literature), Cochrane Central Register of Controlled Trials, and the Cochrane Database of Systematic Reviews were searched. Other databases such as the Chinese Biomedical Literature Database and the Chinese Science Citation Database were not retrieved. As a result, some potentially eligible RCTs which were incorporated into a systematic review and meta-analysis [4] performed by researchers from China are not included in this study.

## Abbreviations

RCT: Randomized controlled trial
SMD: Standardized mean difference
